# Association Between Music Engagement and Mortality in Middle-Aged and Older Adults in the US

**DOI:** 10.1093/geroni/igab046.2288

**Published:** 2021-12-17

**Authors:** Andrew Fiscella, Britney Veal, Ming Ji, Hongdao Meng

**Affiliations:** University of South Florida, Tampa, Florida, United States

## Abstract

Music engagement is a universal human activity that transcends cultural and geographic boundaries. Current evidence suggests that music engages many diverse brain networks with wide-ranging effects on physiological, cognitive, and affective processes. As a result, music activity engagement may be associated with enhanced cognitive reserves and reduced stress. However, it remains unclear whether music activity engagement is associated with any survival advantage in the general population. This study tested this hypothesis in a nationally-representative sample of middle-aged and older adults in the U.S. A cohort of 3,540 respondents from the Health and Retirement Study was followed from 2002 to 2018. Music engagement was measured by self-reported participation in passive and/or active music activities. Potential confounders included socio-demographics, health and functional status, and health-related behaviors. We plotted the Kaplan-Meier survival curves by music engagement level and used Cox proportional hazards model to examine the independent effect of music engagement on mortality. Musical engagement levels were significantly associated with mortality in both the unadjusted and adjusted analyses. Respondents who reported engaging with music at a moderate or high level had lower mortality risk as compared to those who did not (HR=.83, p=0.015; HR=.78, p=0.003, respectively). These findings suggest that music engagement in the middle to late life may have an independent beneficial effect in promoting longevity. Future research should examine whether this observed effect was causal and existed in other populations. If confirmed, interventions should be designed to promote music engagement among middle-aged and older adults.

